# A new species of the genus *Humboldtiana* (Gastropoda, Stylommatophora, Xanthonychidae) from Sierra de Penjamo, Guanajuato, Mexico

**DOI:** 10.3897/BDJ.12.e132797

**Published:** 2024-10-11

**Authors:** Omar Mejía, Benjamín López

**Affiliations:** 1 Laboratorio de Variación Biológica y Evolución, departamento de Zoología, Escuela Nacional de Ciencias Biológicas, Instituto Politécnico Nacional, Mexico, Mexico Laboratorio de Variación Biológica y Evolución, departamento de Zoología, Escuela Nacional de Ciencias Biológicas, Instituto Politécnico Nacional Mexico Mexico; 2 Laboratorio de Variación Biológica y Evolución, departamento de Zoología, Escuela Nacional de Ciencias Biológicas, Instituto Politécnico Nacional, Ciudad de México, Mexico Laboratorio de Variación Biológica y Evolución, departamento de Zoología, Escuela Nacional de Ciencias Biológicas, Instituto Politécnico Nacional Ciudad de México Mexico

**Keywords:** landsnail, taxonomy, Trans-Mexican Volcanic Belt

## Abstract

**Background:**

Mexican terrestrial malacofauna is highly diverse, but poorly studied. The genus *Humboldtiana* includes near to 60 species, most with insular distributions on single mountains from South Texas (USA) to entral Mexico.

**New information:**

*Humboldtianadugesi* sp. nov. is described from the state of Guanajuato, Mexico. This new species represents the first one described for the state and is diagnosed by the following combination of characters that allow distinguishing for other species included in the genus, a protoconch with growth lines and granulated microsculpture, a globose penis that bears a stout verge that covers half of the penis cavity and a long flagellum that is four times longer than the combined length of the penis and epiphallus.

## Introduction

Geographically, Mexico comprises a wide transition zone between the Nearctic and Neotropical Regions, making the country one of the most diverse. Particularly, the Mexican continental gastropod fauna comprises more than 1000 recognised species (López et al. 2022), although it has been estimated that only 25% of the diversity has been described (Thompson 2011). The genus *Humboldtiana* comprises nearly 60 species with an insular distribution in montane habitats from south Texas and New Mexico to the Trans-Mexican Volcanic Belt (Thompson 2011); most of the species are only known from the type locality and only three species could be considered to have a wide distribution, *H.durangoensis*, *H.buffoniana* and *H.nuevoelonis* (Thompson 2006, Mejía et al. 2018). The genus *Humboldtiana* presents a very conservative body-plan in the shell morphology; most of the species have a globose shell covered by granules in most part of the teloconch with three brown bands. The main differences are presented in the reproductive anatomy, leading to the suggestion of a morphostatic radiation (Mejía and Zúñiga 2007). Current systematics of the genus allow it to be split into six subgenera, based both in shell and reproductive anatomy features: H. (Polyomphala) (Thompson and Brewer 2000); H. (Oreades) (Thompson and Brewer 2000); *H. (Gymnopallax)* (Thompson 2006); H. (Clydonacme) (Thompson 2006); *H. (Aglotrochus)* (Thompson 2006); and H. (Humboldtiana) (Ihering 1892. Within the subgenus Humboldtiana, three species groups could be recognised, based on the location of the dart glands; in the first instance, Burch and Thompson (1957) recognised the *H.buffoniana* species group to include all species with dart glands just above the dart sacs and the *H.texana* species group that include all species where the dart glands are separated from the dart sacs by a distance equal to or higher than the maximum length of the dart sacs, posteriorly. Thompson and Brewer (2000) recognised the *H.bicincta* species group to include species where both the dart glands and the dart sacs are reduced. The purpose of this paper is to describe a new species of the *Humboldtianabuffoniana* species group that currently comprises 20 species.

## Materials and methods

Three living snails and two juvenile empty shells were collected by hand amidst rocks and leaf litter. Once in the laboratory, snails were relaxed with menthol and fixed in 70% ethanol. Specimens were studied and dissected using an Olympus stereomicroscope. Reproductive anatomy was hand-drawn and illustrated. The nomenclature and size measurements of the shell, as well as the reproductive anatomy, follows Thompson (2006). Type materials are housed in the Colección Nacional de Malacología at the Instituto de Biología of the Universidad Nacional Autónoma de México, México City. We describe three individuals for which only the holotype was dissected.

## Taxon treatments

### Humboldtiana (Humboldtiana) dugesi

Mejía and López, 2024 sp. nov.

316BD1D6-82BC-5EA3-9954-D96079CBB481

6203FD1C-3DC9-4DCC-B44E-CD2C2F170AD2

#### Materials

- **Type status:** Holotype. **Occurrence:** catalogNumber: CNMO8475; recordedBy: Benjamín López; individualCount: 1; sex: hermaphrodite; lifeStage: adult; establishmentMeans: native; occurrenceStatus: present; preparations: 70% ethanol; disposition: voucher; occurrenceID: FCC03603-7F20-585D-A20D-009531CC693B; **Taxon:** scientificNameID: *Humboldtianadugesi*; acceptedNameUsageID: *Humboldtianadugesi*; parentNameUsageID: *Humboldtiana*; scientificName: *Humboldtianadugesi*; acceptedNameUsage: *Humboldtianadugesi*; parentNameUsage: *Humboldtiana* (Ihering 1892); higherClassification: Animalia | Mollusca | Gastropoda | Stylommatophora | Xanthonychidae |*Humboldtiana* | *Humboldtiana*; kingdom: Animalia; phylum: Mollusca; class: Gastropoda; order: Stylommatophora; family: Xanthonychidae; genus: Humboldtiana; subgenus: Humboldtiana; specificEpithet: *dugesi*; taxonRank: species; scientificNameAuthorship: Mejía and López 2024; nomenclaturalCode: ICZN; taxonomicStatus: accepted; **Location:** continent: North America; country: Mexico; countryCode: MX; stateProvince: Guanajuato; county: Cuerámaro; municipality: Cuerámaro; locality: Fuerte de los remedios, 9 km South of Cuerámaro, Guanajuato; verbatimLocality: 9 km South of Cueramaro, Guanajuato; verbatimElevation: 1939 m; verbatimCoordinates: 20°32.74308'N 101°40.00566'W; verbatimLatitude: 20.545718; verbatimLongitude: -101.666761; verbatimCoordinateSystem: decimal degrees; verbatimSRS: WGS84; decimalLatitude: 20.545718; decimalLongitude: -101.666761; geodeticDatum: WGS84; coordinatePrecision: 0.00001; georeferencedBy: Benjamín López; georeferenceProtocol: GPS; **Identification:** identifiedBy: Omar Mejía and Benjamín López; dateIdentified: 2024; **Event:** samplingProtocol: by hand; samplingEffort: 8 hours; eventDate: 11-01-19; eventTime: 14:00; startDayOfYear: 304; endDayOfYear: 365; year: 2019; month: 11; day: 1; verbatimEventDate: November 2019; habitat: oak forest; **Record Level:** language: en; basisOfRecord: PreservedSpecimen
- **Type status:** Paratype. **Occurrence:** catalogNumber: CNMO8476; recordedBy: Benjamín López; individualCount: 1; sex: hermaphrodite; lifeStage: adult; establishmentMeans: native; occurrenceStatus: present; preparations: 70% ethanol; disposition: voucher; occurrenceID: FCC37484-6CBA-5F2D-BAC1-727172D7EB29; **Taxon:** scientificNameID: *Humboldtianadugesi*; acceptedNameUsageID: *Humboldtianadugesi*; parentNameUsageID: *Humboldtiana*; scientificName: *Humboldtianadugesi*; acceptedNameUsage: *Humboldtianadugesi*; parentNameUsage: *Humboldtiana* (Ihering 1892); higherClassification: Animalia | Mollusca | Gastropoda | Stylommatophora | Xanthonychidae |*Humboldtiana* | *Humboldtiana*; kingdom: Animalia; phylum: Mollusca; class: Gastropoda; order: Stylommatophora; family: Xanthonychidae; genus: Humboldtiana; subgenus: Humboldtiana; specificEpithet: *dugesi*; taxonRank: species; scientificNameAuthorship: Mejía and López 2024; nomenclaturalCode: ICZN; taxonomicStatus: accepted; **Location:** continent: North America; country: Mexico; countryCode: MX; stateProvince: Guanajuato; county: Cuerámaro; municipality: Cuerámaro; locality: Fuerte de los remedios, 9 km South of Cuerámaro, Guanajuato; verbatimLocality: 9 km South of Cueramaro, Guanajuato; verbatimElevation: 1939 m; verbatimCoordinates: 20°32.74308'N 101°40.00566'W; verbatimLatitude: 20.545718; verbatimLongitude: -101.666761; verbatimCoordinateSystem: decimal degrees; verbatimSRS: WGS84; decimalLatitude: 20.545718; decimalLongitude: -101.666761; geodeticDatum: WGS84; coordinatePrecision: 0.00001; georeferencedBy: Benjamín López; georeferenceProtocol: GPS; **Identification:** identifiedBy: Omar Mejía and Benjamín López; dateIdentified: 2024; **Event:** samplingProtocol: by hand; samplingEffort: 8 hours; eventDate: 11-01-19; eventTime: 14:00; startDayOfYear: 304; endDayOfYear: 365; year: 2019; month: 11; day: 1; verbatimEventDate: November 2019; habitat: oak forest; **Record Level:** language: en; basisOfRecord: PreservedSpecimen
- **Type status:** Paratype. **Occurrence:** catalogNumber: CNMO8476; recordedBy: Benjamín López; individualCount: 1; sex: hermaphrodite; lifeStage: adult; establishmentMeans: native; occurrenceStatus: present; preparations: 70% ethanol; disposition: voucher; occurrenceID: 4E828B86-11D0-592B-AE26-4D43D3AE1A78; **Taxon:** scientificNameID: *Humboldtianadugesi*; acceptedNameUsageID: Humboldtianadugesi; parentNameUsageID: *Humboldtiana*; scientificName: *Humboldtianadugesi*; acceptedNameUsage: *Humboldtianadugesi*; parentNameUsage: *Humboldtiana* (Ihering 1892); higherClassification: Animalia | Mollusca | Gastropoda | Stylommatophora | Xanthonychidae |Humboldtiana | Humboldtiana; kingdom: Animalia; phylum: Mollusca; class: Gastropoda; order: Stylommatophora; family: Xanthonychidae; genus: Humboldtiana; subgenus: Humboldtiana; specificEpithet: *dugesi*; taxonRank: species; scientificNameAuthorship: Mejía and López 2024; nomenclaturalCode: ICZN; taxonomicStatus: accepted; **Location:** continent: North America; country: Mexico; countryCode: MX; stateProvince: Guanajuato; county: Cuerámaro; municipality: Cuerámaro; locality: Fuerte de los Remedios, 9 km South of Cuerámaro, Guanajuato; verbatimLocality: 9 km South of Cueramaro, Guanajuato; verbatimElevation: 1939 m; verbatimCoordinates: 20°32.74308'N 101°40.00566'W; verbatimLatitude: 20.545718; verbatimLongitude: -101.666761; verbatimCoordinateSystem: decimal degrees; verbatimSRS: WGS84; decimalLatitude: 20.545718; decimalLongitude: -101.666761; geodeticDatum: WGS84; coordinatePrecision: 0.00001; georeferencedBy: Benjamín López; georeferenceProtocol: GPS; **Identification:** identifiedBy: Omar Mejía and Benjamín López; dateIdentified: 2024; **Event:** samplingProtocol: by hand; samplingEffort: 8 hours; eventDate: 11-01-19; eventTime: 14:00; startDayOfYear: 304; endDayOfYear: 365; year: 2019; month: 11; day: 1; verbatimEventDate: November 2019; habitat: oak forest; **Record Level:** language: en; basisOfRecord: PreservedSpecimen

#### Description

**Shell**

**Figure 1.**
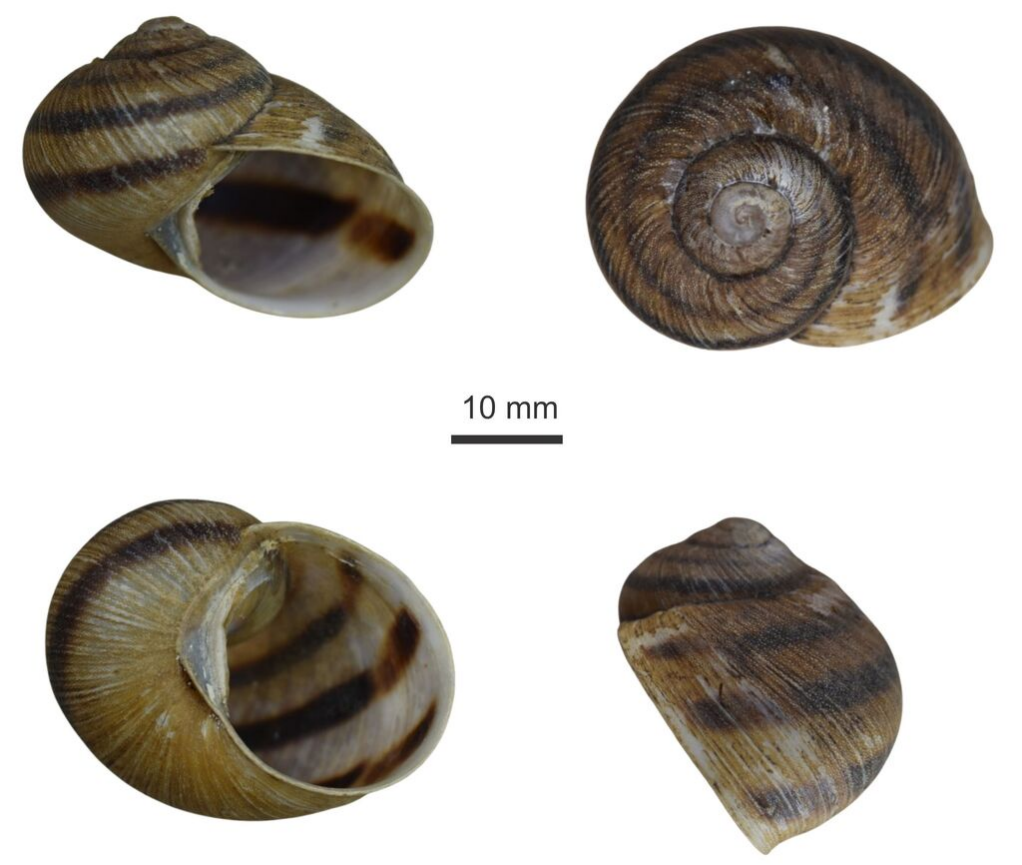
*Humboldtianadugesi* sp. nov. holotype CNMO8475.

**Figure 2.**
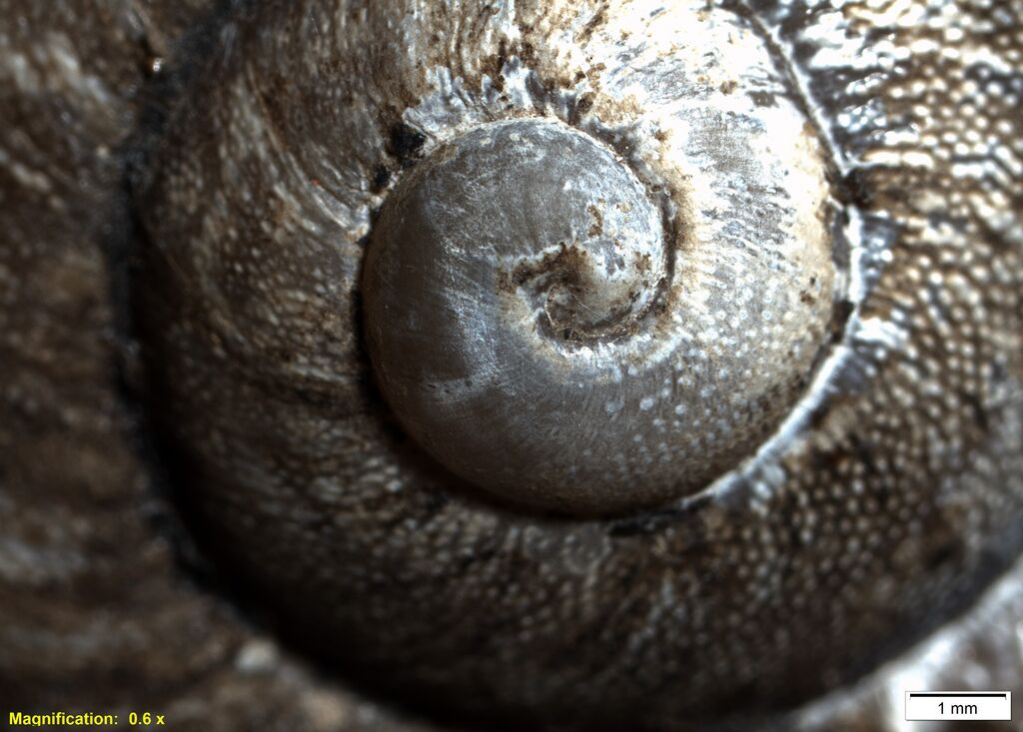
*Humboldtianadugesi* sp. nov. holotype CNMO8475 showing the details of the protoconch.

Description is based on the holotype, the numbers in brackets refer to the paratype I and paratype II, respectively. The shape is globose, the outer lip slightly thickened, pale brown, with three dark-brown bands; in the holotype and paratype I, the third band is wider than the first two; in paratype II, the middle band is the widest; and the other two are barely perceptible. The interior of the shell is white and shiny and the bands are visible. Shell with 4.2 whorls (4.1, 4.0). Protoconch shell caramel in colour, with 1.8 whorls (1.8 whorls, 1.8 whorls); the first 1.3 whorls without sculpture, then some isolated granules from 1.4 to 1.5 and the rest of the protoconch with well-marked growth lines. Sculpture of teleoconch consisting of white growth lines and covered by small oval granules. The umbilicus is obliquely perforate. Parietal callus is thin, translucent and white. Shell height 27.3 mm (27.3 mm, 20.0 mm); shell diameter 36.1 mm (35.5 mm, 26.0 mm); aperture height 19.9 mm (18.0 mm, 14.6 mm); aperture diameter 19.1 mm (19.6 mm, 13.2 mm) (Fig. 1 and Fig. 2).

**Reproductive anatomy**

**Figure 3.**
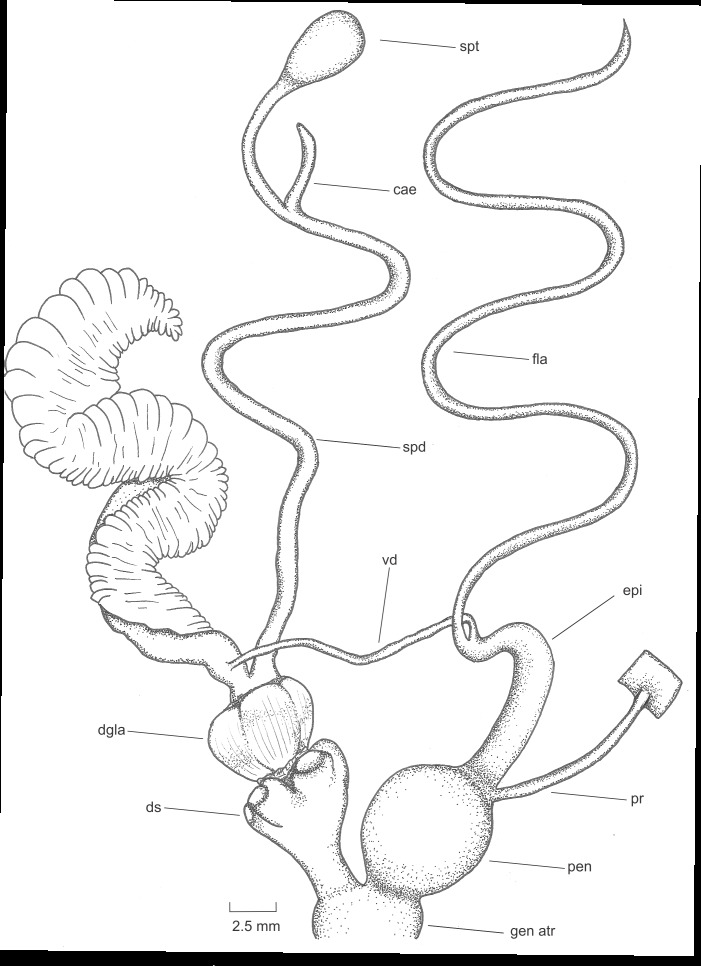
Reproductive anatomy of *Humboldtianadugesi* sp. nov. holotype CNMO8475. Abbreviations: cae, spermathecal caecum; dgla, dart glands; ds, dart sacs; epi, epiphallus; fla, flagellum; gen atr, genital atrium; pen, penis; pr, penis retractor; spd, spermathecal duct; spt, spermatheca; vag, vagina; vd, vas deferens.

Penis globose, 6.9 mm, interior of the penis with a stout verge extending half of the penis cavity. The penis retractor muscle is inserted at the base of the epiphallus, which is 11 mm long. The epiphallus is short, cylindrical and measures 10.9 mm. Flagellum long, 73 mm in length, is nearly 4.07x the combined length of the penis plus epiphallus. The genital atrium is short, measuring 2.5 mm. The lower vagina is short and slightly taller than half penis size, 3.65 mm, extending to the region of the dart sacs; four dart sacs approximately of the same size, 2 mm. The median vagina bears four dart glands, forming a ring just above the dart sacs, with the dart glands reaching a maximum height of 3.2 mm. The spermathecal duct is 52 mm in length, the spermatheca bears a caecum of 5.4 mm length and it adheres to the albumen gland, the spermatheca is elongated and sac-shaped, with a length of 4.7 mm (Fig. 3).

#### Diagnosis

*Humboldtianadugesi* sp. nov. can be distinguished from other species included in the subgenus Humboldtiana by a protoconch with growth lines and granulated microsculpture, in opposition to the rest of the species of the subgenus where the protoconch lacks sculpture. On the other hand, in the reproductive anatomy, *H.dugesi* shared with *H.potosiana* the presence of a globose penis, but in *H.dugesi*, the penis bears a stout verge that covers half of the penis cavity; also, in *H.dugesi*, the flagellum is long, 4x the combined length of the penis plus epiphallus; meanwhile, in *H.potosiana*, the flagellum is short, barely 1x the combined length of the penis plus epiphallus.

#### Etymology

This species is dedicated to the French-Mexican naturalist of the 19^th^ century, Alfredo Dugès, a pioneer in the study of natural history in Mexico, particularly in the state of Guanajuato.

#### Distribution

Mexico: Guanajuato; Cuerámaro; Sierra de Penjamo; Fuerte de los Remedios, only known from type locality.

#### Ecology

Three individuals were collected active in rock crevices during the day, two empty shells were collected in leaf litter, at an altitude of 1939 m a.s.l. The vegetation of the type locality is a temperate subhumid oak forest.

#### Taxon discussion

**Figure 4.**
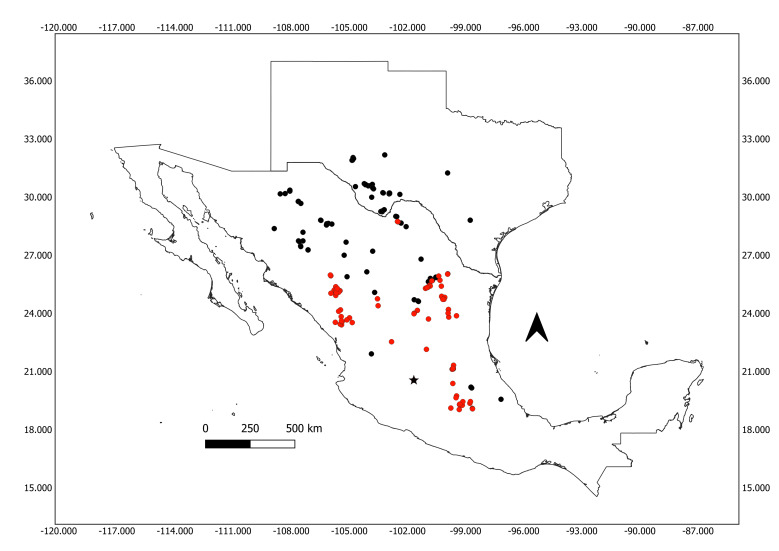
Geographic distribution of the species included in the genus *Humboldtiana*. The star represents the collection record of *Humboldtianadugesi* sp. nov. described in this paper. For details of the collection records of each species, please see the Supplementary file. Records of the species included in the *Humboldtianabuffoniana* species group are marked in red.

The new species described in this paper could be assigned to the *Humboldtianabuffoniana* species group of the subgenus Humboldtiana by the almost smooth sculpture of the protoconch and the dart glands just above the dart sacs. The Humboldtiana (Humboldtiana) buffoniana group comprises 20 species distributed from the border between New Mexico and Texas (*Humboldtianaultima*) to the Mexican Transvolcanic Belt (Fig. 4). Since most of the species of the genus show an insular distribution and are only known from the type locality, we only compare the new species to the four closely-geographically distributed species of the Humboldtiana (Humboldtiana) buffoniana group, *Humboldtianapotosiana* Pilsbry, 1927 from Sierra de San Miguelito, San Luis Potosí (Pilsbry 1927); *H.salviahispanica* (Mejía et al. 2009) from Huichapan, Hidalgo, *H.queretaroana* (Dall 1897) and *H.pinicola* (Thompson and Brewer 2000), both from the Municipality of Pinal de Amoles in the Sierra Gorda of Querétaro (Fig. 4, Suppl. material 1). *Humboldtianadugesi* has a small shell that is similar to *H.salviahispanica* and *H.potosiana*; also, the number of whorls and shell sculpture are similar; however, *H.dugesi* can be diagnosed by the presence of growth lines and isolated granules in the protoconch, the last of which is a unique character within the genus. On the other hand, in the reproductive anatomy, *H.dugesi* shares with *H.potosiana* a globose penis, but in *H.dugesi*, the epiphallus is long and cylindrical, the flagellum is long and 4x the combined length of the penis + epiphallus and the spermathecal appendix is present. In contrast, in *H.potosiana*, the epiphallus is short and stout, the flagellus is short, barely 1x the combined length of the penis + epiphallus and the spermathecal appendix is absent (Table 1).

## Supplementary Material

XML Treatment for Humboldtiana (Humboldtiana) dugesi

1F6CF336-D136-59E9-8269-275B7FFD1D18 10.3897/BDJ.12.e132797.suppl1 Supplementary material 1 Collection records of the species currently recognised in the genus Humboldtiana Data type occurrences Brief description The table contains the collection records of all currently recognised species of the genus *Humboldtiana* including the geographic coordinates of *Humboldtianadugesi* sp. nov. described in this paper. File: oo_1085301.xlsx https://binary.pensoft.net/file/1085301 Omar Mejía and Benjamín López-López

## Figures and Tables

**Table 1.** Species included in the *Humboldtianabuffoniana* species group used for comparison with the new species.

Character	*H.dugesi* holotype	*H.salviahispanica*	*H.pinicola*	*H.potosiana*	*H.queretaroana*
Shell size	small 27 mm height	small 22 mm height	large 38 mm height	small 24 mm height	large 37 mm height
Number of whorls	4.2	4.1-4.2	4.4-4.8	4.2	5
bands' colour pattern	dark brown visible at the interior	chestnut to dark brown visible at the interior	black visible at the interior	dark brown with a reddish tinge	absent
Shell sculpture	growth lines and granules	growth lines and granules	growth lines and granules	growth lines and granules	growth lines and granules
embryonic shell whorls	1.8	1.5-1.6	1.6-1.7	1.5	1.5
embryonic shell sculpture	1.3 smooth, then isolated granules and growth lines	1.0 smooth then almost imperceptible growth lines	smooth	smooth	smooth
Penis	globose 6.9 mm	short and stocky 7.8 mm	short and stocky 9. 6 mm	globose	NA
verge	stout covering half penis cavity	large and broadened composed of two lobes	long and extending to opening vagina with three or four lobes	NA	NA
epiphallus	long and cylindrical 11 mm	long and cylindrical 16 mm	short and stout 7 mm	short and stout	NA
flagellum	long 73 mm	short 35 mm	short 36 mm	short	NA
flagellum/ penis + epiphallus	4.07 times	1.68 times	1.7-2.6 times	maybe 1 times	NA
dart size length	2 mm	1.5 mm	four darts of unequal size	NA	NA
glands height	3.2 mm	2 mm	NA	NA	NA
spermathecal duct length	52 mm	40 mm	36 mm	NA	NA
spermathecal caecum length	5.4 mm	5 mm	5.4 mm	caecum absent	NA
spermathecal length	4.7 mm	7 mm	25 mm	NA	NA
Note: *Humboldtianaqueretaroana* is only known from the original description (Dall 1897). In *H.potosiana*, the size of the organs is not available (NA).					
